# The Effectiveness of Mindfulness-Based Interventions in the Perinatal Period: A Systematic Review and Meta-Analysis

**DOI:** 10.1371/journal.pone.0155720

**Published:** 2016-05-16

**Authors:** Billie Lever Taylor, Kate Cavanagh, Clara Strauss

**Affiliations:** 1 Sussex Partnership NHS Foundation Trust, Brighton, United Kingdom; 2 School of Psychology, University of Sussex, Brighton, United Kingdom; Carnegie Mellon University, UNITED STATES

## Abstract

Perinatal mental health difficulties are associated with adverse consequences for parents and infants. However, the potential risks associated with the use of psychotropic medication for pregnant and breastfeeding women and the preferences expressed by women for non-pharmacological interventions mean it is important to ensure that effective psychological interventions are available. It has been argued that mindfulness-based interventions may offer a novel approach to treating perinatal mental health difficulties, but relatively little is known about their effectiveness with perinatal populations. This paper therefore presents a systematic review and meta-analysis of the effectiveness of mindfulness-based interventions for reducing depression, anxiety and stress and improving mindfulness skills in the perinatal period. A systematic review identified seventeen studies of mindfulness-based interventions in the perinatal period, including both controlled trials (n = 9) and pre-post uncontrolled studies (n = 8). Eight of these studies also included qualitative data. Hedge’s *g* was used to assess uncontrolled and controlled effect sizes in separate meta-analyses, and a narrative synthesis of qualitative data was produced. Pre- to post-analyses showed significant reductions in depression, anxiety and stress and significant increases in mindfulness skills post intervention, each with small to medium effect sizes. Completion of the mindfulness-based interventions was reasonable with around three quarters of participants meeting study-defined criteria for engagement or completion where this was recorded. Qualitative data suggested that participants viewed mindfulness interventions positively. However, between-group analyses failed to find any significant post-intervention benefits for depression, anxiety or stress of mindfulness-based interventions in comparison to control conditions: effect sizes were negligible and it was conspicuous that intervention group participants did not appear to improve significantly more than controls in their mindfulness skills. The interventions offered often deviated from traditional mindfulness-based cognitive therapy or mindfulness-based stress reduction programmes, and there was also a tendency for studies to focus on healthy rather than clinical populations, and on antenatal rather than postnatal populations. It is argued that these and other limitations with the included studies and their interventions may have been partly responsible for the lack of significant between-group effects. The implications of the findings and recommendations for future research are discussed.

## Introduction

Pregnancy and the period following childbirth are critical times for women, which are associated with substantial emotional and physical changes. While many women find these changes largely positive, for some they can render them vulnerable to mental health difficulties [1]. The most extensively researched mental health problem in the perinatal period (defined here as the time from the onset of pregnancy up to the end of an infant’s first year) is depression. Period prevalence estimates suggest that nearly a fifth of women experience depression during pregnancy and a similar proportion do so in the first three months after giving birth [2]]. Anxiety disorders in the perinatal period have received less attention than depression, but a large population-based study in the US reported that 13% of pregnant or postpartum women had experienced an anxiety disorder in the past year [3]. While some studies have suggested that the incidence of mental health difficulties in the perinatal period is not significantly elevated compared with other times in a woman’s life [4, 5], others have found an increased risk once unequal distribution of risk factors between perinatal and non-perinatal populations is controlled for [6]. In addition, it is not only mothers who are at risk of mental health problems: around 10% of fathers also experience depression in the perinatal period [7], compared to a 12-month prevalence rate of 4.8% for depression in the male population as a whole [8].

Perinatal mental health difficulties are associated with serious adverse consequences for parents and infants. For example, severe postpartum psychiatric disorders are associated with high rates of maternal suicide, with a seventy-fold increase in suicide risk in the first postnatal year compared to age-specific rates for the general female population [9]. There is also evidence that perinatal mental health difficulties are associated with poorer pregnancy outcomes [10] and longstanding emotional, social and cognitive difficulties in children, which appear to be mediated by problematic early parent-infant interactions [11]. Research suggests there are significant cost implications too: in the United States women with postpartum depression have been shown to incur 90% higher health services expenditure than non-depressed postpartum women [12], while in the UK it has been estimated that perinatal depression, anxiety and psychosis together carry with them a total long-term cost to society of around £8.1 billion for each one-year cohort of births [13]. Effective treatment of mental health problems during pregnancy and postnatally is therefore vital and it has been pointed out that the perinatal period also offers a key opportunity for interventions aimed at building resilience and preventing poor mental health [1].

In the UK the National Institute for Health and Clinical Excellence (NICE) guidelines point out that: “There are risks associated with taking psychotropic medication in pregnancy and during breastfeeding” ([14], p.4) and research shows that many women express a preference for psychological interventions during the perinatal period, expressing fears about medication harming their developing baby, guilt about taking medication, and concerns about becoming dependent [15]. This means that identifying effective therapies is particularly important [16] and NICE guidelines recommend psychological interventions, often in the form of cognitive-behavioural therapy (CBT), for women with perinatal depression or anxiety wherever the likely benefits outweigh the risks of medication [14].

A recent meta-analysis of CBT for perinatal depression found significantly greater reductions in symptoms of depression for women receiving CBT than for controls [17]. Nevertheless, mean effect sizes were only small to medium (*g* = 0.39 for prevention studies and g = 0.65 for treatment studies), and around a quarter of participants (23%) dropped out of studies on average. This suggests that, although potentially effective for some, CBT may not be a panacea. Indeed a previous meta-analysis of psychological interventions for postnatal depression, which examined the effectiveness of a range of interventions such as CBT, Interpersonal Psychotherapy (IPT), counselling and social support interventions reported smaller effect sizes for depression than those typically found with these interventions in adult populations, with no indication of differing effect sizes by intervention type (where this could be measured) type [18]. Furthermore, the authors failed to find evidence of significant effects in the longer term, although they acknowledged that this could have been because of the small number of studies examining longer term effects. Meanwhile, very few studies have investigated the effectiveness of CBT for anxiety disorders in perinatal women, and those that have done so have not always found significant benefits of CBT compared with control conditions [19]. Taken as a whole, these findings suggest it is important to further examine whether alternative psychological therapies may be effective for perinatal difficulties.

Mindfulness-based interventions (MBIs) are relatively novel psychological approaches that have potential in perinatal care [20]. Mindfulness has been defined as “the awareness that emerges through paying attention on purpose, in the present moment, and non-judgementally to the unfolding of experience moment by moment” [21], p.145. The most commonly available and researched MBIs, considered as the ‘gold standard’, are mindfulness-based stress reduction (MBSR) and mindfulness-based cognitive therapy (MBCT). These are eight week group interventions involving 2–3 hour weekly group sessions and an additional all-day session. In MBSR and MBCT participants engage in mindfulness practices of up to 30–40 minutes in the group sessions followed by teacher-led group discussion. Daily home mindfulness practice is also encouraged and supported through audio recordings. Mindfulness practices are verbally guided and invite participants to focus attention with an attitude of curiosity and acceptance on present-moment experiences such as the breath, sensations in the body, sounds and thoughts. A meta-analysis of six randomised controlled trials found that MBCT was effective in comparison to control conditions at reducing the relative risk of depressive relapse by 43% in people who were currently well but had a history of three or more episodes of depression [22], and MBCT is recommended for this population by UK national clinical guidelines [23].

Given that MBCT was specifically designed to prevent depression in those at risk, it has been argued that it may be of value in the perinatal period where, as outlined, the risk of developing depression or experiencing a depressive relapse may be elevated [20].

There is also good empirical evidence that MBIs can reduce current symptoms of depression, anxiety and stress [24–28], suggesting they may be valuable where such difficulties do emerge. Recent evidence suggests that MBIs work in part by reducing levels of rumination and, given that factors such as ‘brooding rumination’ have been found to predict the maintenance of depression in the perinatal period [30], mindfulness may be a particularly apt intervention at this time.

Hughes et al also speculate that MBIs may improve early parent-infant interactions by increasing parents’ ability to attend to their infants without becoming preoccupied by negative or self-critical thoughts, and may help women relate to pain differently thereby reducing anxiety associated with childbirth.

This suggests that MBIs may have potential in the perinatal period both to prevent the onset of and to alleviate existing mental health difficulties. However, while MBCT and MBSR and generally considered to be ‘gold standard’ MBIs, there is diversity in the way MBIs have been adapted for and targeted at perinatal populations, with MBIs often being of shorter duration, involving briefer mindfulness practices than in MBCT or MBSR and/or requiring fewer or briefer home practices. To date, there has only been one published review of mindfulness training in the perinatal period [31]. This review found evidence from some studies of pre- to post-MBI improvements in stress, anxiety and depression in pregnant women but between-group differences in the two randomised controlled trials (RCTs) on these outcomes were non-significant. The authors did not set out to combine findings in a meta-analysis and, by their own admission, their conclusions are limited. This is compounded by the fact that the sample sizes of the two included RCTs were too small to detect anything other than large effects–that is, failure of the RCTs to find significant between-group differences could simply be due to the studies being underpowered. In addition, the review focused exclusively on antenatal rather than postnatal populations and also did not include analysis of several recent randomised-controlled trials of MBIs (e.g. [32, 33, 34, 35, 36, 37]). Therefore, it remains uncertain as to whether or not the evidence to date suggests that MBIs are effective for perinatal populations and this question would benefit from a meta-analysis which could comprehensively draw together and summarise this evidence.

This paper therefore presents a systematic review and meta-analysis of the effectiveness of MBIs in the perinatal period. Uncontrolled and controlled trials of MBIs for perinatal populations were reviewed and Hedges *g* was calculated with the aim of exploring the pre-post and post-intervention between-group impact of MBIs on depression, anxiety, stress and mindfulness skills. Analyses were also carried out to examine whether variables including number of sessions with a therapist, study quality, baseline symptom severity, and primary study target (e.g. depression, anxiety, or stress) moderated (i.e. predicted) the effects. A narrative synthesis of qualitative data was also produced.

## Method

### Literature search

Studies were identified through searches of electronic databases from inception to 28^th^ February 2016, including PsychInfo, ProQuest, Web of Science, Scopus and the Cochrane Library.

All articles including the terms “mindful*”, “MBCT” or “MBSR” in combination with “perinatal”, “prenatal”, “antenatal”, “postnatal”, “postpartum”, “puerperal”, “pregnancy”, “pregnant”, “trimester”, “childbirth”, “child”, “baby”, “babies”, “infant”, “offspring”, “mother”, “father”, “maternal”, “paternal” or “parent” in the title were identified. Reference lists of retrieved papers were also searched manually to identify additional potentially eligible studies, and one additional paper known to the authors was included.

### Eligibility criteria

To be included in the review: 1) papers had to be published in a peer-reviewed journal in English; 2) studies could be qualitative or quantitative but had to explore the effectiveness of an MBI during the perinatal period (i.e. during pregnancy or the first year following childbirth), with MBIs defined as MBCT or MBSR or an intervention described by the author(s) as based on mindfulness practices and principles; 3) quantitative studies were required to collect data at baseline and after the intervention; 4) quantitative outcomes had to include validated self-report or clinician-administered measures of depression, anxiety, stress and/or mindfulness. No methodological requirements were set, although study quality was rated (see Section 2.4).

Studies of Acceptance and Commitment Therapy (ACT) [38] were excluded. These were not included because, although mindfulness forms part of ACT, it is not possible to separate out the effectiveness of mindfulness from the effectiveness of ACT in its entirety. Studies exploring mindfulness for mothers who had experienced infant mortality or stillbirth were excluded. Where more than one paper was identified relating to the same sample, these were reviewed in combination.

### Data extraction and analysis

Means, standard deviations (SDs) and number of participants were extracted at baseline and post-intervention for measures of depression, anxiety, stress and mindfulness (baseline or pre-test outcomes were defined as those obtained prior to the intervention commencing and post-test or post-intervention outcomes were defined as those taken directly after completion of the intervention). http://journals.plos.org/plosone/article?id=10.1371/journal.pone.0096110-pone.0096110-Cochrane1 Intention-to-treat (ITT) data were entered where available, otherwise observed cases data were included (see Table 1 for details). Authors were contacted where data for the primary outcome was not available within the study reports (data was obtained from four studies in this way). In line with other meta-analyses of the effectiveness of psychological interventions in the perinatal period [17] if more than one measure of depression was available, the Edinburgh Postnatal Depression Scale (EPDS [39]) was selected wherever possible. The EPDS is the most frequently used self-report measure of perinatal depressive symptom severity with strong psychometric properties amongst both pregnant and postpartum samples [39, 40]. For other outcomes (i.e. anxiety and stress), or in cases where the EPDS was not administered, where more than one outcome measure of the construct was used the measure with the strongest psychometric properties was selected. Where mindfulness was measured by the Five Facet Mindfulness Questionnaire (FFMQ, [41]), the total score was used. Where this was not available (e.g. if only certain subscales were used) the non-judge facet was selected since this subscale has been found to have the highest associations with measures of depression, anxiety and stress [41, 42].

**Table 1:** Characteristics of included studies (*n = 17*).

Authors	Location and design	Participants (n = final sample)	Intervention details and mindfulness practice (1 = frequency and duration of in-session mindfulness practice; 2 = frequency and duration of between-session mindfulness practice)	Delivery	Intervention duration	Outcome measures				Engagement	Quality rating
						Depression	Anxiety	Stress	Mindfulness		/9
Barber et al (2013) [64]	New Zealand. Uncontrolled pre-post study. Qualitative data also collected at post-intervention via interview.	Pregnant women recruited from the community (n = 8)	Computerised self-help mindfulness and relaxation programme. Biofeedback used to reinforce learning. Aimed to reduce stress and distress. (1: not reported; 2: not reported).	Antenatal online	15 steps completed at participants’ own pace (approx. 45 minutes per step).	EPDS	STAI-S	PSS	MAAS	n = 9 enrolled. 100% completion rate, although one participant admitted only superficial engagement and was excluded from the analysis.	1
Beddoe et al (2009) [65]	US. Uncontrolled pre-post study.	Healthy women pregnant with first baby (n = 16)	Mindfulness-based yoga. Based on lyengar yoga and MBSR. Aimed to reduce distress. (1: mindfulness practice in each session (duration of in-session practice not reported);2: not reported).	Antenatal group	7 weekly group sessions, 75mins each	N/A	STAI-S	PSS	FFMQ-NJ	n = 19 recruited. n = 2 (11%) dropped out of the intervention; in addition n = 1 excluded due to missing data.	4
Byrne et al (2013) [63];Fisher et al (2012) [66]	Australia. Uncontrolled pre-post study with follow-up approximately 3–12 weeks post-birth. Qualitative data gathered at follow-up through focus groups.	Healthy pregnant women (pre-post: n = 12; follow-up: n = 16)	Mindfulness-Based Childbirth Education (MBCE). Aimed to reduce fear of childbirth/anxiety and stress and increase self-efficacy. Also attended by birth partners. (1: not reported; 2: daily, but duration of home practices not reported).	Antenatal group	8 weekly group sessions, 2.5hrs each	EPDS/; DASS-D*	DASS-A	DASS-S	MAAS	n = 18 (100%) completed the full programme. n = 6 excluded from pre-post analysis due to missing data, n = 2 excluded from follow-up data due to missing data.	3
Chan (2015) [32]	Hong Kong. RCT (control group received introductory lecture only). Follow-up data reported at around 5 weeks postpartum.	Convenience sample of pregnant women. Intervention (n = 64); control (n = 56)	Eastern Based Meditative Intervention (EBMI). Aimed to reduce distress and improve wellbeing/coping. (1: not reported; 2: daily, but duration of home practices not reported).	Antenatal group	6 sessions, length and frequency not stated	EPDS	N/A	N/A	N/A	Of 179 participants recruited, 59 (33%) were excluded due to missing data. Attrition from intervention not reported.	1
Dimidjian et al (2015) [56]	US. Uncontrolled pre-post study. Depression measured at baseline and post-intervention, at each MBCT session, and then monthly up to 6 months postpartum. Post-intervention interview.	Pregnant women with prior major depressive disorder, but not currently clinically depressed (n = 37)	Adapted Mindfulness Based Cognitive Therapy (MBCT). Aimed at prevention of depression. (1: not reported; 2: home practice at least 6 days per week. N.B. the authors noted that intervention adhered to the standard MBCT intervention manual though with some modifications).	Antenatal group	8 weekly sessions, 2hrs each. Option of monthly follow-up class	EPDS	N/A	N/A	N/A	88% (n = 43) completed at least 50% of the intervention. Average of 6.1 sessions attended out of 8. N = 37 provided post-intervention EPDS data.	3
Dimidjian et al (2016) [37]	US. RCT (treatment as usual control). Depression measured at baseline and post-intervention, at each MBCT session, and then monthly up to 6 months postpartum.	Pregnant women with prior major depressive disorder, but not currently clinically depressed. Intervention (n = 24); control (n = 31)	Adapted Mindfulness Based Cognitive Therapy (MBCT). Aimed at prevention of depression. (1: not reported; 2: home practice at least 6 days per week; N.B. the authors noted that intervention adhered to the standard MBCT intervention manual though with some modifications).	Antenatal group	8 weekly sessions, 2hrs each. Option of monthly follow-up class	EPDS	N/A	N/A	N/A	Of the 37 participants who started MBCT-PD, 33 (89%) completed at least 50% of the intervention. Average of 6.89 sessions completed. N = 24 intervention participants provided post-intervention EPDS data.	6
Duncan & Bardacke (2010) [67]	US. Uncontrolled pre-post study. Qualitative feedback post-birth via interviews.	Community sample of pregnant women (n = 27)	Mindfulness-Based Childbirth & Parenting. Aimed to improve maternal wellbeing. Attended by birth partners as well. (1: mindfulness practice in each session (duration of in-session practice not reported); 2: home practice 6 days per week (30 mins per practice)).	Antenatal group	9 weekly sessions, 3 hrs each (plus 7hr silent retreat and post-birth reunion)	CES-D	PAS	PSS	FFMQ-NJ	n = 35 signed up. n = 8 (23%) dropped out (mostly as gave birth before completing intervention). Average attendance for those attending >1 session was 8.3/10	3
Dunn et al (2012) [62]	Australia. Intervention group and non-randomised treatment-as-usual control group. Data collected at pre, post and 6-weeks after birth. Qualitative data collected post-birth via interviews.	Pregnant women outpatients at an antenatal clinic. Pre-post: intervention (n = 4); control (n = 4). Follow-up: intervention (n = 4); controls (n = 4)	Antenatal Adapted MBCT. Not explicitly promoted as a treatment for mental health (although this was implicit). (1: not reported; 2: not reported. N.B. authors state that intervention adhered to the standard MBCT treatment manual but with small adaptions).	Antenatal group	8 weekly sessions, session duration not stated	EPDS	DASS-A	DASS-S	MAAS	n = 14 registered for treatment. n = 3 (21%) did not attend any sessions. Only n = 4 intervention participants provided post-intervention data and n = 6 provided follow-up data.	4
Gambrel & Piercy (2013) [33]; Gambrel & Piercy (2015) [68]	US. RCT (waitlist control). Qualitative post-intervention feedback also collected via interview.	Couples expecting first baby (either pregnant or adopting). Intervention (n = 32; 17 women including one lesbian couple; 15 men); Control (n = 34; 17 women, 17 men)	Mindful Transition to Parenthood Program. Aimed primarily at improving couple relationship. (1: not reported; 2: daily home practice, 15 mins per practice).	Antenatal group	4 weekly sessions, 2hrs each	DASS-D	DASS-A	DASS-S	FFMQ	n = 36 intervention participants completed pre-measures. n = 4 (11%) attended < = 1 session. Remainder attended at least 3 of 4 sessions.	4
Goodman et al (2005) [59]	US. Uncontrolled pre-post study. Qualitative feedback collected at post-intervention.	Pregnant women with GAD/significant anxiety symptoms (PSWQ< = 45 or BAI> = 11 or met criteria for GAD on MINI) (n = 23).	Antenatal Coping with Anxiety through Living Mindfully (CALM) pregnancy intervention. Based on MBCT. Aimed to reduce perinatal anxiety. (1: meditation practice in each session (duration of in-session practice not stated); 2: daily home practice with 30–45 mins per practice).	Antenatal group	8 weekly 2 hr sessions	BDI-II	BAI	N/A	MAAS	n = 26 enrolled. n = 3 (12%) attended <3 sessions and did not complete post-assessment. n = 23 (88%) completed at least 50% of sessions.	3
Guardino et al (2014) [60]	US. RCT (active control; received pregnancy book). Follow-up at 6-week post-intervention.	Pregnant women with scores >34 on the PSS or >1 on the PSA. Intervention (n = 24); control (n = 23). Intent-to-treat analysis.	Mindful Awareness Practices (MAPS). Aimed at reducing stress. Attended by general public as well. (1: mindfulness practice in each session (duration of in-session practice not stated); 2: daily, 5–17 mins per practice).	Antenatal group	6 weekly 2hr classes	N/A	STAI-S	PSS	FFMQ	n = 24 randomised. n = 17 (71%) attended four or more of the six sessions.	6
Miklowitz et al (2015) [58]	US. Uncontrolled pre-post study, 1 and 6 month post-intervention follow-up.	Pregnant, trying to conceive or up to one year postpartum. Lifetime diagnosis of major depression or bipolar spectrum disorder. Current subthreshold symptoms of depression (n = 39).	Aimed at reducing depression. (1: mindfulness practice in each session (duration of in-session practice not stated); 2: usually 45 mins daily).	Group (women attending could be antenatal, postnatal, or trying to conceive)	8 weekly sessions, 2 hrs each	BDI-II	N/A**	N/A	N/A**	n = 49 completed baseline. n = 46 invited to MBCT intervention. n = 39 enrolled and completed at least one session. n = 32 (65%) completed at least 4 sessions.	4
Muzik et al (2012) [57]	US. Uncontrolled pre-post study. Brief qualitative post-intervention feedback.	Pregnant women (first baby) scoring > = 9 on EPDS (n = 18).	Antenatal Mindfulness yoga (M-Yoga). Aimed to improve mood. (1: yoga practice in each session accompanied by descriptions of ‘mindfulness qualities’–e.g. “practice the pose for your body without; judgment”; 2: home practice encouraged–frequency/duration not reported).	Antenatal group	10 weekly sessions, 90 minutes each	EPDS	N/A	N/A	FFMQ	n = 22 recruited. n = 2 (9%) dropped out after first session. In addition, n = 2 did not complete post-measures.	2
Perez-Blasco et al (2013) [34]	Spain. RCT (no intervention control).	Breastfeeding mothers (57.1% were first-time mothers; average baby age 10.75 months). Intervention (n = 13); control (n = 8)	Based on MBCT, MBSR and Mindful self-compassion. Aimed at distress, well-being and self-efficacy. Babies in the room. (1: mindfulness practice in each session (2–3 x 10 min meditations per session); 2: 2 x 20 min home practices per day).	Postnatal group	8 weekly sessions, 2hrs long	DASS-D	DASS-A	DASS-S	FFMQ	All intervention participants completed post-intervention measures. Attrition from intervention not stated.	3
Vieten & Astin (2008) [35]	US. RCT (waitlist control). Follow-up data collected 3 months post-intervention.	Community sample of pregnant women who had previously sought treatment for “mood concerns” Intervention (n = 13); control (n = 18)	Mindful Motherhood. Aimed to improve stress and mood, and regulate distressing affect. (1: mindfulness practice within sessions (not stated if in every session or of what duration); 2: 3 x 20 minute daily home practice).	Antenatal group	8 weekly group sessions, 2hrs long	CES-D	STAI-S	N/A	MAAS	n = 15 enrolled in intervention. n = 2 (13%) dropped out. Participants attended a mean of 7.2 of 8 sessions.	5
Woolhouse et al (2014) [61]	Australia. RCT (treatment as usual control). Additional uncontrolled pre-post study arm. Qualitative data collected post-intervention via interviews.	For RCT sample of healthy pregnant women recruited via range of avenues through their contact with antenatal clinic. Intervention (n = 13); control (n = 10). For uncontrolled pre-post study, sample included pregnant women deemed by antenatal clinic to be at risk of depression or anxiety (n = 11)	MindBabyBody. Aimed to reduce depression, anxiety and stress. (1: mindfulness practice in each session (duration of in-session practice not stated); 2: daily home practice with 15–20 mins per practice).	Antenatal group	6 weekly sessions, 2hrs each	CES-D	STAI-S	PSS	FFMQ	RCT: n = 17 enrolled in intervention group. n = 4 (24%) did not complete post-data (n = 3 delivered baby prior to completing intervention; n = 1 did not do questionnaire). Uncontrolled study: n = 20 enrolled. n = 9 (45%) did not complete post-data (n = 4 withdrew; n = 4 gave birth prior to completing intervention; n = 1 did not complete questionnaire).	43
Zhang & Emory (2015) [36]	US. RCT (treatment-as-usual control). Pre-post and one-month post-intervention follow-up.	Pregnant African-American women recruited from a hospital or pregnancy programme. Intervention (n = 16); control (n = 17). Intent-to-treat analysis.	Mindful Motherhood. Aimed to improve stress and mood, regulate distressing affect and help mothers be more present with their infants. (1: not reported; 2: not reported).	Antenatal group	8 sessions over 4 weeks, 2hrs each	BDI-II	N/A	PSS	Toronto Mindfulness Scale	n = 34 assigned to intervention condition. Only 9% (n = 3) of intervention participants completed the eight session programme.	6

BAI = Beck Anxiety Inventory; BDI-II = Beck Depression Inventory; CES-D = Center for Epidemiologic Studies Depression Scale; DASS-D/A/S = Depression Anxiety and Stress Scale–short form, depression (D), anxiety (A) or stress (S) subscale; EPDS = Edinburgh Postnatal Depression Scale; FFMQ = Five Factor Mindfulness Scale; GAD = General Anxiety Disorder Scale; MINI = Mini Mental State Examination; MAAS = Mindful Attention Awareness Scale; PAS = Pregnancy Anxiety Scale; PSA = Pregnancy Specific Anxiety Scale; PSS = Perceived Stress Scale; PSWQ = Penn State Worry Questionnaire; STAI-S = State Trait Anxiety Inventory, state subscale.

*DASS-D reported in pre to follow-up analysis as EPDS only administered pre and post

** This study did also measure anxiety and mindfulness but means and standard deviations for these measures were not provided and thus they were not included in the analysis.

To measure methodological quality, an index was required that could accommodate non-randomised studies as well as randomised trials. However, while a number of quality assessment tools exist, and have been used for evaluating uncontrolled studies, most omit key quality domains and all have weaknesses [43]. After exploring a range of options, it was considered most appropriate for the purposes of this review to use a quality index developed for a recent large-scale comprehensive meta-analysis of mindfulness-based therapy which included both randomised and non-randomised studies [44]. This included items from the Jadad scale [45] along with additional items not specific to controlled studies. The index included the following items: (1) whether intervention followed a clearly described protocol based on, or adapted from, an established programme (i.e. MBCT or MBSR) (score of 0 or 1); (2) whether measures were administered at follow-up (score of 0 or 1); (3) whether a validated measure of mindfulness was included (score of 0 or 1); (4) whether it was reported that therapists were trained in delivering mindfulness-based therapy *and* (for studies with clinical populations only) were clinically trained (based on good practice guidelines for teaching mindfulness-based courses [46], this item was adapted to specify that mindfulness training was required for any study to obtain a score of 1, but clinical training was only required for studies including clinical populations) (score of 0 or 1); (5) whether the study was randomised (score of 0 if not randomised, 1 if randomised with a no intervention/waitlist control, 2 if randomised with a intervention as usual control, and 3 if randomised with an active control); (6) whether evaluators and/or participants were blinded regarding condition (score of 0 if not blinded, 1 if single-blinded, 2 if double-blinded). Final scores were out of 9, with higher scores reflecting studies of higher quality.

Hedge’s *g* pre-post effect sizes were calculated for all studies (including RCTs) for depression, anxiety, stress and mindfulness outcomes. Hedges’s *g* is a variation of Cohen’s *d* that corrects for biases due to small sample sizes [47]. It is calculated as the difference in the mean scores for the chosen outcome measure, divided by the within-groups standard deviation (which can be computed from the standard deviation of the difference scores) and adjusted to take account of small sample sizes. The formula used to calculate Hedge’s *g* was:

$$g=\frac{{\overset{-}{Y}}_{1}-{\overset{-}{Y}}_{2}}{S_{within}}\;\left(1-\frac{3}{4df-1}\right)$$

$$S_{within}=\frac{S_{diff}}{\sqrt{2(1-r)}}$$

$$S_{diff}=\sqrt{{{sd}_{1}}^{2}+{{sd}_{2}}^{2}-2r{sd}_{1}{sd}_{2}}$$

where ${\overset{-}{Y}}_{1}$ is the pre-intervention sample mean, ${\overset{-}{Y}}_{2}$ is the post-intervention sample mean, *S*_*within*_ is the within-groups standard deviation, *df* is the degrees of freedom, *S*_*diff*_ is the standard deviation of the difference scores, *r* is the correlation between pre and post-intervention scores, *sd*_1_ is the standard deviation of the pre-intervention mean, and *sd*_2_ is the standard deviation of the post-intervention mean. As the correlation *r* was typically not available within the study reports, the recommendation by Rosenthal [48] was followed and a conservative estimation of *r* = 0.7 assumed.

For controlled studies, post-intervention between-group effect sizes were also calculated using Hedges *g*. In this case, Hedges *g* is the between-group difference on the post-intervention mean scores for the chosen outcome measure divided by the within-groups standard deviation, pooled across groups (roughly speaking, the average standard deviation of the two groups) and adjusted to take account of small sample sizes. Hedges *g* was calculated using the following formula:

$$g=\frac{{\overset{-}{X}}_{1}-{\overset{-}{X}}_{2}}{S_{within}}\left(1-\frac{3}{4df-1}\right)$$

$$S_{within}=\sqrt{\frac{(n_{1}-1){{sd}_{1}}^{2}+(n_{2}-1){{sd}_{2}}^{2}}{n_{1}+n_{2}-2}}$$

where ${\overset{-}{X}}_{1}$ is the post-intervention mean on the chosen outcome measure for group 1; ${\overset{-}{X}}_{2}$ is the post-intervention mean on the chosen outcome measure for group 2; *n*_1_ is the number of participants in group 1; *n*_2_ is the number of participants in group 2; *sd*_1_ is the standard deviation of the post-intervention mean for group 1; and *sd*_1_ is the standard deviation of the post-intervention mean for group 2.

The magnitude of Hedges’s g can be interpreted using Cohen’s [49] convention as small (0.2), medium (0.5), or large (0.8).

For the meta-analyses, the inverse variance method was used to calculate overall effect sizes, with weights applied to studies based on the following formula:

$$W=\frac{1}{{SE}^{2}}$$

where *SE* is the standard error. For pre-post studies, *SE* was calculated as:

$$SE=\sqrt{\frac{1}{n}+\frac{d^{2}}{2n}\;2(1-r)}\;\left(1-\frac{3}{4df-1}\right)$$

where *n* is the number of pairs of data. For between-groups studies, *SE* was calculated as:

$$SE=\sqrt{\frac{n_{1+}n_{2}}{n_{1}n_{2}}+\frac{d^{2}}{2(n_{1+}n_{2})}}\;\left(1-\frac{3}{4df-1}\right)$$

A random effects model was used for the meta-analyses, estimated via the method of moments. The pre-post meta-analysis was conducted using SPSS macros provided by Wilson [50]. The between-group meta-analysis was conducted with Review Manager version 5.2. Tables or forest plots of effect sizes were produced for each outcome variable. Where effects were significant, funnel plots were produced and Rosenthal’s failsafe N was calculated to explore publication bias.

In order to assess the extent to which effect sizes were significantly different from each other homogeneity was assessed using the Q statistic. A significant Q value means there is significant heterogeneity within the effect sizes, which, where relevant, was explored using moderation analyses. Moderator analyses attempt to identify predictors of heterogeneous effects and these were planned using ANOVA and regression meta-analytic analogues with the number of sessions with a therapist and study quality entered as predictors into separate regression analyses to test their association with the effects of the MBIs. Baseline symptom severity (high versus low) and primary study target (i.e. depression, anxiety, or stress/general distress/wellbeing) were also planned as a moderator analyses using ANOVA.

## Results

**Fig 1:**
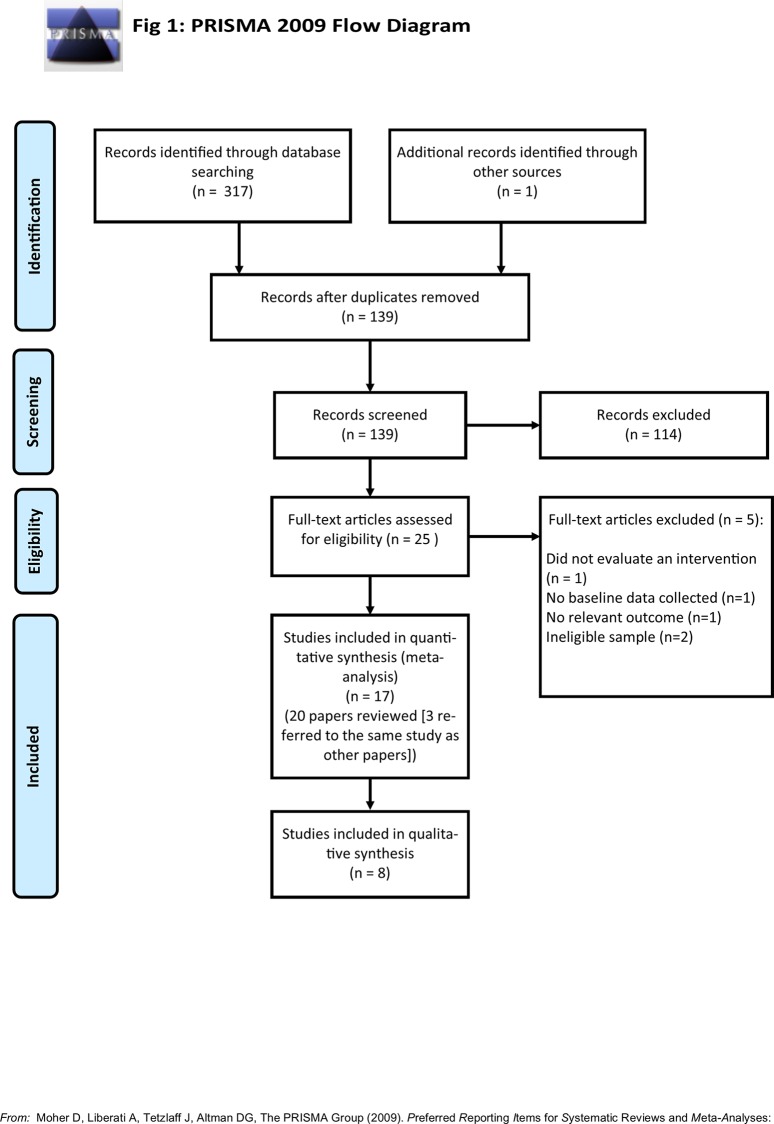
PRISMA diagram of search results.

Fig 1 shows a PRISMA flow diagram of the search results. In total, 139 articles were screened after removing duplicates, and twenty-five full-text articles were accessed (the remainder were excluded as screening of titles and/or abstracts revealed they did not meet the eligibility criteria outlined in section 2.1). One study was excluded because it did not evaluate an intervention [51], one did not include baseline data [52], two were excluded because they did not include eligible samples of women [53, 54], and one did not include a relevant outcome measure [55]. Seventeen separate studies were included in the review (one of these studies included two separate samples of pregnant women). One study included breastfeeding mothers with infants from newborn up to two years old (rather than just up to one year old). However, it was included as it was nevertheless clearly designed to target the early postnatal period and the mean baby age was under one year (10.75 months).

### Characteristics of included studies

Table 1 provides a summary of the included studies and their key characteristics.

Only one study related to an intervention carried out postnatally, while one intervention was carried out with a mixture of pregnant women, postpartum women and women actively trying to conceive. The other fifteen studies all related to MBIs carried out during pregnancy. In all but one study, the intervention used a group-based format (ranging from four to ten weeks in duration): the other study evaluated a fifteen-step computerised intervention. Two studies focused primarily on the prevention of depression (in samples of pregnant women who were currently well but had a history of major depression [37, 56]. One study focused on reducing current depression in pregnant women with elevated depressive symptoms [57], one focused on improving mood in women with a history of treatment for “mood concerns” [35], and one focused on improving depressive symptoms in women with a history of major depression or bipolar spectrum disorder and current subthreshold depressive symptoms [58]. Two studies focused on reducing anxiety or stress in pregnant women with elevated anxiety and/or stress symptoms [59; 60], while the uncontrolled arm of Woolhouse et al’s, [61] study recruited women deemed to be at risk of stress, anxiety or depression by their midwives or obstetricians. The remaining studies focused on reducing general distress or stress or improving wellbeing, sometimes in conjunction with a focus on reducing symptoms of depression or anxiety, in samples of participants not selected on the basis of having elevated scores on baseline measures. This was with the exception of one study which focused primarily on improving couples’ relationships with each other but also examined mental health outcomes.

As outlined in Table 1, the seventeen included studies administered a wide variety of different mindfulness interventions: only four studies explicitly reported following the standard MBCT training manual albeit with modifications in some cases such as customising mindfulness practices for the perinatal period [37; 56; 58; 62]. Ten further studies stated that they were based at least partly on either MBCT or MBSR, while three did not refer to having a basis in either MBCT or MBSR. In twelve of the seventeen studies it was reported that participants were encouraged to undertake mindfulness practice at home at least 6 days per week, although, where reported, recommended practices were sometimes briefer in duration than recommended in MBCT or MBSR (see Table 1). Moreover, where reported, the frequency and duration of actual home practice was often considerably less than recommended. Finally, in addition to teaching mindfulness, in some cases interventions included additional elements such as childbirth education (e.g. [63]) and psychoeducation about the transition to parenthood (e.g. [33]).

All seventeen studies reported quantitative data, and eight also included qualitative feedback from participants (one paper reported qualitative data only but as this shared its sample with a quantitative study it was reviewed in conjunction with the quantitative study). Eight studies used a randomised-controlled trial (RCT) design (one of these also included a separate uncontrolled pre-post study arm). Six of the eight RCTs used a waitlist, no intervention, or treatment-as-usual control condition, while one had an active control group (in which participants read a pregnancy book) and one included an introductory mindfulness lecture for control participants. One study had a non-randomised control group. Eight used an uncontrolled pre-post design. In total, nine studies included additional quantitative follow-up data (i.e. data collected not only post-intervention but also at an additional later time point), although one of these did not report means and standard deviations for follow-up data and therefore was excluded from the analysis (one additional study included qualitative rather than quantitative follow-up data). One study reported data for male as well as female participants, while the remainder included only women in the analysis.

In terms of study quality, scores out of nine ranged from one to six, with a mean quality score of four. Quality scores were lowered by the fact that the majority of studies were uncontrolled

Overall, analysis included data from 595 participants, including 32 men (5.4%) and 563 women (94.6%). Final sample sizes in the study analyses ranged from 8 to 120 participants.

### Pre-post effects

Of the 17 included studies, 15 included pre and post data for a measure of depression (total n = 354), 11 for anxiety (total n = 193), 11 for stress (total n = 189), and 13 for mindfulness (total n = 227). There were significant small to medium pre to post improvements for each of these outcomes. The mean Hedges’s *g* pre-post effect size estimate was *g =* 0.47 (95% CI [0.33, 0.60], p < .01) for reducing depression, *g* = 0.36 (95% CI [0.17, 0.56], p < .01) for reducing anxiety, *g* = 0.49 (95% CI [0.27, 0.71], p < .01) for reducing stress, and *g* = 0.51 (95% CI [0.38, 0.65], p < .01) for increasing mindfulness skills (see Tables 2–5). Heterogeneity was significant for all outcomes (*Q* = 37.34, 39.35, 41.72, 25.51 respectively, all p < .05). For those studies that included follow-up data, effects at follow-up time points remained significant for depression (*g* = 0.36, 95% CI [0.21, 0.50], p < .01), anxiety (*g* = 0.64, 95% CI [0.32, 0.95], p < .01), stress (*g* = 0.56, 95% CI [0.19, 0.94, p < .01], and mindfulness (*g* = -0.65, 95% CI [-0.87, -0.44] p < .01). However, as only three to six studies were included in these follow-up analyses, these results must be treated with caution.

**Table 2:** Pre-post effect sizes for depression (*n =* 15).

Authors		Pre		Post		Hedge’s *g*, 95% CI
	*n*	M	SD	M	SD	
Barber et al (2013) [64]	8	9.25	3.69	5.38	2.62	1.02 [0.41, 1.63]
Byrne et al (2013) [63]	12	7.33	5.07	7.00	2.83	0.06 [-0.35, 0.47]
Chan (2015) [32]	64	7.95	3.58	6.77	3.79	0.32 [0.12, 0.52]
Dimidjian et al (2015)* [56]	37	5.82	4.61	3.38	3.02	0.56 [0.31, 0.81]
Dimidjian et al (2016)* [37]	24	5.98	3.95	4.67	3.95	0.32 [0.01, 0.63]
Duncan & Bardacke (2010) [67]	27	1.63	0.45	1.48	0.34	0.35 [0.06, 0.64]
Dunn et al (2012) [62]	4	13.00	7.35	9.25	6.85	0.39 [-0.22, 1.00]
Gambrel & Piercy (2013) [33]	1517	5.073.29	5.182.11	2.402.94	3.043.09	0.52 [0.13, 0.91] (men)0.12 [-0.23, 0.47] (women)
Goodman et al (2005) [59]	23	11.87	5.67	6.39	6.36	0.87 [0.50, 1.24]
Miklowitz et al (2015) [58]	39	12.20	12.30	7.40	7.00	0.41 [0.16, 0.66]
Muzik et al (2012)* [57]	20	12.45	3.41	7.60	4.16	1.20 [0.77, 1.63]
Perez-Blasco et al (2013) [34]	13	4.46	2.60	2.31	2.56	0.78 [0.33, 1.23]
Vieten & Astin (2008)* [35]	13	20.40	8.40	16.20	7.30	0.49 [0.08, 0.90]
Woolhouse et al (2014) [61]	1012	14.4224.60	10.058.19	12.0818.20	4.179.13	0.22 [-0.17, 0.61] (RCT arm pre-post)0.67 [0.18, 1.16] (uncontrolled arm)
Zhang & Emory (2015) [36]	16	18.90	11.20	17.30	10.20	0.14 [-0.21, 0.49]
**Overall total (weighted)**	**354**					**0.47 [0.33, 0.60]**

*Depression was primary outcome

**Table 3:** Pre-post effect sizes for anxiety (*n* = 11).

Authors		Pre		Post		Hedge’s *g*, 95% CI
	*n*	M	SD	M	SD	
Barber et al (2013) [64]	8	32.88	11.50	30.75	6.99	0.18 [-0.31, 0.67]
Beddoe et al (2009) [65]	88	26.7030.40	5.4012.10	31.4031.90	16.009.00	-0.25 [-0.74, 0.24] (2^nd^ trimester women)-0.12 [-0.61, 0.37] (3^rd^ trimester women)
Byrne et al (2013)* [63]	12	8.33	7.57	6.00	7.53	0.29 [-0.12, 0.70]
Duncan & Bardacke (2010) [67]	27	2.49	0.58	2.09	0.41	0.73 [0.42, 1.04]
Dunn et al (2012) [62]	4	10.50	14.36	6.00	8.16	0.25 [-0.34, 0.84]
Gambrel & Piercy (2013) [33]	1517	6.134.59	5.733.73	4.535.18	2.774.19	Men: 0.27 [-0.10, 0.64]Women: -0.14 [-0.49, 0.21]
Goodman et al (2005)* [59]	23	12.13	8.56	6.35	4.95	0.70 [0.35, 1.05]
Guardino et al (2013) [60]	24	45.69	7.64	39.47	6.27	0.84 [0.49, 1.19]
Perez-Blasco et al (2013) [34]	13	7.08	7.19	2.46	3.38	0.62 [0.19, 1.05]
Vieten & Astin (2008) [35]	13	43.80	12.40	35.40	9.10	0.69 [0.24, 1.14]
Woolhouse et al (2014) [61]	129	35.9249.67	14.1115.22	32.8339.33	7.088.26	0.21 [-0.20, 0.62] (RCT arm pre-post)0.65 [0.14, 1.16] (uncontrolled arm)
**Overall total (weighted)**	**193**					**0.36 [0.17, 0.56]**

*Anxiety was primary outcome

**Table 4:** Pre-post effect sizes for stress (*n* = 11).

Authors		Pre		Post		Hedge’s *g*, 95% CI
	*n*	M	SD	M	SD	
Barber et al (2013) [64]	8	18.75	6.25	13.63	3.62	0.78 [0.21, 1.35]
Beddoe et al (2009) [65]	88	14.0015.40	9.706.90	13.9010.30	12.206.60	0.01 [-0.46, 0.48] (2^nd^ trimester women)0.67 [0.12, 1.22] (3^rd^ trimester women)
Byrne et al (2013) [63]	12	9.83	5.42	11.50	6.45	-0.26 [-0.67, 0.15]
Duncan & Bardacke (2010) [67]	27	26.41	6.73	24.11	4.99	0.36 [0.07, 0.65]
Dunn et al (2012) [62]	4	23.00	7.75	16.00	8.49	0.64 [-0.03, 1.30]
Gambrel & Piercy (2013) [33]	1517	12.0010.47	7.866.65	5.339.65	3.444.20	0.82 [0.39, 1.25] (men)0.13 [-0.22, 0.48] (women)
Guardino et al (2013) [60]	24	41.81	6.00	37.30	5.38	0.76 [0.43, 1.09]
Perez-Blasco et al (2013) [34]	13	18.31	4.31	9.54	6.44	1.38 [0.81, 1.95]
Vieten & Astin (2008) [35]	13	20.10	5.10	15.90	5.70	0.72 [0.27, 1.17]
Woolhouse et al (2014) [61]	1311	17.9222.46	7.145.79	16.5417.18	6.125.84	0.19 [-0.2, 0.58] (RCT arm pre-post)0.84 [0.33, 1.35] (uncontrolled arm)
Zhang & Emory (2015) [36]	16	43.90	10.20	39.70	7.46	0.42 [0.05, 0.79]
**Overall total (weighted)**	**189**					**0.51 [0.30, 0.72]**

**Table 5:** Pre-post effect sizes for mindfulness skills (*n* = 13).

Authors		Pre		Post		Hedge’s *g*, 95% CI
	*n*	M	SD	M	SD	
Barber et al (2013) [64]	8	4.00	0.79	4.33	0.77	-0.38 [-0.89, 0.13]
Beddoe et al (2009) [65]	88	30.9030.20	10.1010.20	33.0035.00	15.004.50	-0.14 [-0.63, 0.35] (2^nd^ trimester participants)-0.43 [-0.94, 0.08) (3^rd^ trimester participants)
Byrne et al (2013) [63]	12	4.04	0.84	4.47	0.51	-0.51 [-0.94, -0.08]
Duncan & Bardacke (2010) [67]	27	3.50	0.57	3.78	0.60	-0.46 [-0.75, -0.17]
Dunn et al (2012) [62]	4	51.25	16.09	57.00	15.21	-0.27 [-0.86, 0.32]
Gambrel & Piercy (2013) [33]	1517	103.60102.82	10.1213.52	113.00107.35	11.498.65	-0.81 [-1.24, -0.38] (men)-0.35 [-0.70, 0.00] (women)
Goodman et al (2005) [59]	23	51.04	9.50	54.87	10.62	-0.36 [-0.67, -0.05]
Guardino et al (2013) [60]	24	119.64	13.04	134.24	15.48	-0.97 [-1.34, -0.60]
Muzik et al (2012) [57]	18	131.17	14.23	137.56	16.79	-0.39 [-0.74, -0.04]
Perez-Blasco et al (2013) [34]	13	27.62	4.56	34.15	3.87	-1.43 [-2.02, -0.84]
Vieten & Astin (2008) [35]	13	3.60	0.76	3.80	0.82	-0.24 [-0.63, 0.15]
Woolhouse et al (2014) [61]	1110	121.55116.55	23.6513.27	134.55130.73	20.5519.83	-0.53 [-1.00, -0.06] (RCT arm pre-post) -0.72 [-1.21, -0.23] (uncontrolled arm)
Zhang & Emory (2015) [36]	16	30.10	11.10	35.10	6.78	-0.46 [-0.83, -0.09]
**Overall total (weighted)**	**227**					**-0.51 [-0.87, -0.44]**

When only measures corresponding to the primary outcome (i.e. the intervention target) for the intervention were included in the pre-post analysis (i.e. depression measures for interventions targeting depression, anxiety measures for interventions targeting anxiety, and stress measures for interventions targeting stress/general distress/wellbeing), the overall effect size was *g* = 0.56 (95% CI [0.42, 0.70], p < .01). Heterogeneity was again significant (*Q* = 35.90, p < .01).

#### Moderation of pre-post effects

As heterogeneity was significant in the pre-post analyses, moderator analyses were performed. Significantly larger effect sizes were found for the eight studies that recruited participants with elevated symptoms of anxiety, depression or stress (or a history of these difficulties) than for studies that recruited healthy pregnant women, for both anxiety outcomes (Hedge’s *g* = 0.73 versus *g =* 0.22, between-groups *Q* = 8.51, p < .01) and depression outcomes (Hedge’s *g* = 0.61 versus *g* = 0.35, between-groups *Q* = 4.41, p < .05). There were no significant moderation effects of this variable for stress or mindfulness outcomes (between-groups *Q* range = 0.01–2.02, p>.05).

There were no significant differences in effect sizes for studies depending on whether the primary outcome of the intervention was depression, anxiety or stress/general distress/wellbeing (between-groups *Q* = 0.07, p = .967).

The number of sessions with a therapist did not significantly predict pre-post effect sizes for depression (β = 0.03, p = .883), anxiety (β = 0.40, p = .123), stress (β = -0.15, p = .593) or mindfulness (β = 0.09, p = .718). Likewise, study quality did not predict pre-post effect sizes for depression (β = -0.35, p = .144), anxiety (β = 0.29, p = .308), stress (β = -0.09, p = .751) or mindfulness (β = -0.20, p = .417).

#### Publication bias

**Fig 2:**
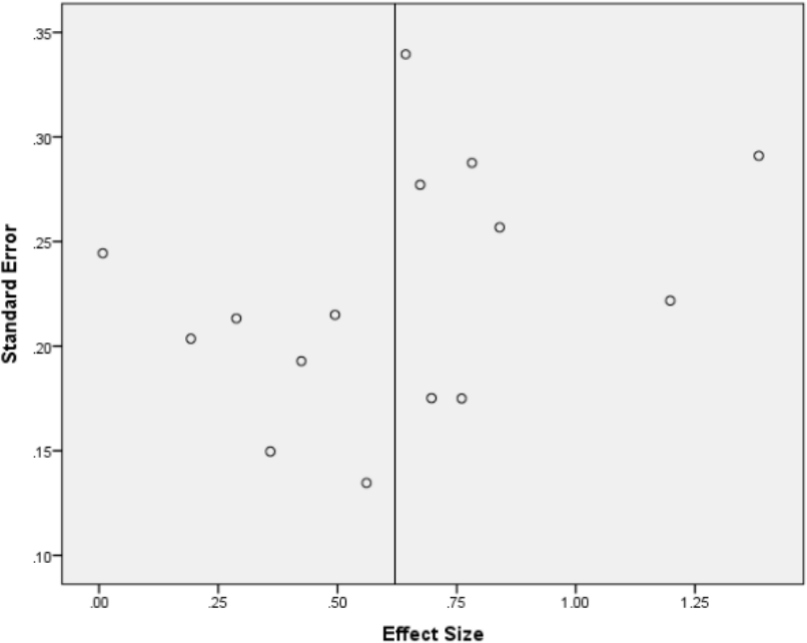
Funnel plot of effect sizes by standard error for primary outcome.

As Fig 2 shows, a funnel plot of the pre-post effect sizes for the primary outcome against the related standard errors gave some indication of publication bias, suggesting that smaller studies were associated with larger effect sizes. Nevertheless, Rosenthal’s fail safe N for the pre-post effect sizes of the primary outcome was 157, which means 157 unpublished studies with null results would have to be included for this effect size to be non-significant at the p < .05 level.

### Between-group effects

Tables 6–9 show tables of results for between-group effects for controlled studies including a measure of depressive symptoms (eight studies; total n = 356), anxiety symptoms (six studies; total n = 194), stress symptoms (seven studies; total n = 229) and mindfulness skills (seven studies; total n = 227). A negative effect for depression, anxiety or stress suggests results favour the intervention group, whereas for mindfulness a positive effect favours the intervention group. There were negligible effect sizes and no significant differences at post-intervention between the intervention and control conditions in depressive symptoms (z(8) = 0.68, *g* = -0.07, 95% CI [-0.28 to 0.14], p = .5); anxiety symptoms (z(6) = 0.14, *g* = -0.02, 95% CI [-0.35 to 0.31], p = .89) and stress symptoms (z(7) = 0.93, *g* = -0.16, 95% CI [-0.49 to 0.17], p = .35), and a small non-significant difference in mindfulness skills (z(7) = 1.08, *g* = 0.22, 95% CI [-0.18 to 0.62], p = .28). Effect sizes were not significantly heterogeneous (p>.05), with the exception of mindfulness skills (χ2(7) = 14.66, p = .04).

**Table 6:** Controlled effects for depression (*n* = 8).

Authors		Experimental			Control		Hedge’s *g*, 95% CI
	*n*	M	SD	*n*	M	SD	
Chan (2015) [32]	64	6.77	3.79	56	6.50	3.32	0.07 [-0.28, 0.43]
Dimidjian et al (2016) [37]	24	4.67	3.95	31	6.39	3.81	-0.44 [-0.98, 0.10]
Dunn et al (2012) [62]	4	9.25	6.85	4	5.75	3.69	0.55 [-0.88, 1.99]
Gambrel & Piercy (2013) [33]	1517	2.402.94	3.043.09	1717	3.414.35	4.236.13	-0.26 [-0.96, 0.43]-0.28 [-0.96, 0.39]
Perez-Blasco et al (2013) [34]	13	2.31	2.56	8	3.50	3.96	-0.36 [-1.25, 0.53]
Vieten & Astin (2008) [35]	13	16.20	7.30	18	17.20	7.40	-0.13 [-0.85, 0.58]
Woolhouse et al (2014) [61]	12	12.08	4.17	10	10.10	8.72	0.29 [-0.56, 1.13]
Zhang & Emory (2015) [36]	16	17.30	10.20	17	15.20	7.70	0.23 [-0.46, 0.91]
**Overall total (weighted)**	**178**			**178**			**-0.07 [-0.28, 0.14]**

**Table 7:** Controlled effects for anxiety (*n* = 6).

Authors		Experimental			Control		Hedge’s *g*, 95% CI
	*n*	M	SD	*n*	M	SD	
Dunn et al (2012) [62]	4	6.00	8.16	4	8.00	5.89	-0.24 [-1.64, 1.15]
Gambrel & Piercy (2013) [33]	1517	4.535.18	4.944.19	1717	3.414.94	3.863.94	0.32 [-0.38, 1.02]0.06 [-0.61, 0.73]
Guardino et al (2013) [60]	24	39.47	6.27	23	37.35	11.51	0.23 [-0.35, 0.80]
Perez-Blasco et al (2013) [34]	13	2.46	3.38	8	7.25	4.40	-1.21 [-2.18, -0.24]
Vieten & Astin (2008) [35]	13	35.4	9.1	18	35.6	8.4	-0.02 [-0.74, 0.69]
Woolhouse et al (2014) [61]	12	32.83	7.08	9	33.0	12.78	-0.02 [-0.88, 0.85]
**Overall total (weighted)**	**98**			**96**			**-0.02 [-0.35, 0.31]**

**Table 8:** Controlled effects for stress (*n* = 7).

Authors		Experimental			Control		Hedge’s *g*, 95% CI
	*n*	M	SD	*n*	M	SD	
Dunn et al (2012) [62]	4	16.0	8.49	4	10.5	8.23	0.57 [-0.87, 2.01]
Gambrel & Piercy (2013) [33]	1517	5.339.65	3.444.20	1717	9.4111.41	7.85.91	-0.65 [-1.36, 0.07]-0.34 [-1.01, 0.34]
Guardino et al (2013) [60]	24	37.3	5.38	23	35.8	8.01	0.22 [-0.36, 0.79]
Perez-Blasco et al (2013) [34]	13	9.54	6.44	8	18.0	8.14	-1.14 [-2.10, -0.18]
Vieten & Astin (2008) [35]	13	15.90	5.70	18	16.90	4.60	-0.19 [-0.91, 0.52]
Woolhouse et al (2014) [61]	13	16.54	6.12	10	14.4	8.41	0.29 [-0.54, 1.12]
Zhang & Emory (2015) [36]	16	39.70	7.46	17	38.90	8.62	0.10 [-0.59, 0.78]
**Overall total (weighted)**	**115**			**114**			**-0.16 [-0.49, 0.17]**

**Table 9:** Controlled effects for mindfulness (*n* = 7).

Authors		Experimental			Control		Hedge’s *g*, 95% CI
	*n*	M	SD	*n*	M	SD	
Dunn et al (2012) [62]	4	57.00	15.21	4	71.25	7.27	-1.04 [-2.60, 0.52]
Gambrel & Piercy (2013) [33]	1517	113.00107.35	11.498.65	1717	112.88107.59	15.1210.57	0.01 [-0.69, 0.70]-0.02 [-0.70, 0.65]
Guardino et al (2013) [60]	24	134.24	15.48	23	134.66	20.17	-0.02 [-0.59, 0.55]
Perez-Blasco et al (2013) [34]	13	34.15	3.87	8	24.63	5.48	2.02 [0.91, 3.13]
Vieten & Astin (2008) [35]	13	3.80	0.82	18	3.60	0.72	0.26 [-0.46, 0.97]
Woolhouse et al (2014) [61]	11	134.55	20.55	10	133.5	12.43	0.06 [-0.80, 0.92]
Zhang & Emory (2015) [36]	16	35.10	6.78	17	31.10	9.94	0.46 [-0.24, 1.15]
**Overall total (weighted)**	**113**			**114**			**0.22 [-0.18, 0.62]**

### Qualitative feedback

Eight studies included qualitative feedback from participants alongside the quantitative data. Findings are summarised in Table 10. Feedback was typically positive. More specifically, participants reported that they thought they benefited from connecting with others within a group context, and thought that they had become more able to focus on the present moment, to regulate their negative responses to difficult situations, and to become more accepting of current experiences. In the only study to include the views of men as well as women, Gambrel and Piercy, 2013 [33, 68] found that while women in the sample typically initiated participation in the intervention, they commented that they already had adequate social support and did not find that the intervention provided additional benefit. Men on the other hand said they had found participating in the intervention valuable reporting that it helped them understand their partners’ experiences of pregnancy better, become more connected with their babies, and identify more strongly with the role of father.

**Table 10:** Summary of qualitative findings.

Authors	Data collection and analytic method	Interviewer	No. interviews as proportion of intervention participants	Key findings
Barber et al (2013) [64]	Qualitative data collected at post-intervention via interview. Analysed using thematic analysis	Not stated	15/15	Participants reported that they found the intervention relaxing and liked completing it before going to sleep. Two had some complaints about the course content (e.g. that it was ‘mumbo-jumbo’) but in general participants appeared positive, and several who already had children reported that it helped them moderate their reactivity to challenging behaviour.
Byrne et al (2013) [63]; Fisher et al (2012) [66]	Qualitative data gathered at follow-up through two focus groups carried out around four months after the intervention—one with women who took part in the intervention and the other with their birth partners. Analysed using thematic analysis	Researchers (not those facilitating the intervention)	12/18 mothers and an additional 7 birth partners	Participants enjoyed the sense of community the group provided. They reported that the intervention helped them recognise their potential, and empowered them by enabling them to gain the confidence to express their wishes during pregnancy and birth, and by helping them remain calm and in control. A number of participants reported continuing to find mindfulness beneficial postnatally.
Dimidjian et al (2015) [56]	Post-intervention interview. Analysed using thematic coding/content analysis	Study evaluators (not stated who these were)	Not stated	Over three quarters of participants (78%) felt the course had been helpful, while 83% said it changed how they coped with intense emotions. Participants also reported that the course helped them: relate differently to depression; identify and respond to triggers and warning signs; and disengage from negative thinking.
Duncan & Bardacke (2010) [67]	Qualitative feedback post-birth via interviews. Analysed using interpretative phenomenological analysis.	Not stated	Not stated	Majority of participants reported that they continued to formally practise mindfulness. Many said they had found learning to stay in the present moment helpful for labour and birth, while others found bringing mindful presence to interactions with babies and partners valuable, along with bringing mindful awareness to emotional reactivity.
Dunn et al (2012) [62]	Qualitative data collected post-birth via interviews. Thematic analysis	Researcher who did not facilitate the intervention	Not stated	All participants reported continuing to practise mindfulness at least informally. Similar to the other studies participants valued connecting with others in a group setting, and found it helpful learning to stay in the present, to notice but not act on thoughts and feelings, and to become more accepting of things as they are.
Gambrel & Piercy (2013) [33]; Gambrel & Piercy (2015) [68]	Qualitative post-intervention feedback also collected via interview. Phenomenological analysis	Researcher who also facilitated the intervention	15/16 couples interviewed (couples interviewed together)	Although women in the sample had typically initiated participation in the intervention, they often commented that they were already receiving adequate support from others. Men on the other hand said they were receiving little support and found connecting with others in the programme valuable. Men also reported that the programme helped them understand their partners’ experiences of pregnancy better, become more connected with their babies, and identify more strongly with the role of father. Women appreciated their partners’ increased understanding, but felt they already naturally identified with the role of mother through their pregnancy.
Muzik et al (2012) [57]	Brief qualitative post-intervention feedback	Self-completed feedback survey	18/22	Participants enjoyed the social support offered by the group, and found the intervention beneficial both during their pregnancy and labour.
Woolhouse et al (2014) [61]	Qualitative data collected post-intervention via interviews. Analysed with interpretative phenomenological analysis.	Researchers (not those who facilitated the intervention)	4/37	Participants initially found the group setting uncomfortable, but ultimately enjoyed it. Most found it challenging to engage in daily mindfulness practice. However, overall they found the intervention helpful for mood, quality of life and sleep as well as for encouraging them to step back and not get caught up in negative emotions, thoughts or behaviours.

### Engagement

Of the seventeen included studies, nine reported on the number of participants meeting a study defined criterion for engagement or completion (completing all fifteen steps [64]; completing the programme [36,63]; completing at least 50% of sessions [37, 56, 58, 59], attending at least three out of four sessions [33]; and completing at least four out of six sessions [60]. In these nine studies, 285 participants were allocated to the intervention condition and 210 (74%) met study defined engagement or completing criteria. Five studies instead reported the number of participants who dropped out of the intervention. In these studies, 128 participants were allocated to the intervention condition and 25 (20%) dropped out. Comparing these levels of engagement with other perinatal interventions is difficult because of inconsistency and lack of clarity around how attrition is reported, but they appear to be in line with the average drop-out rate of 23% reported for CBT for perinatal depression [17].

The frequency of home practice completed by participants was often not recorded and therefore it was difficult to ascertain how much practice participants completed. In traditional MBCT and MBSR courses, participants are asked to practice for 30–45 minutes per day, six days per week. In the studies included in this meta-analysis where home practice was reported, participants typically did not practise as much as this. For example, in Gambrel and Piercey’s, 2013 [33] study, participants were asked to practise for 15 minutes per day, 6 days per week, but did only around half as much as this. In Guardino et al’s[61] study, participants reported practising on 3–4 days per week for 9–10 minutes per day. In Byrne et al ‘s study [63] participants reported practising an average of 3.6 times per week for a total average of 103 minutes over the eight-week intervention. Similarly, Woolhouse et al[61] found that participants in their study most often practised 3–4 days per week, typically for 6–10 minutes at a time. Finally, Dimidjian et al [37, 56] asked participants to practice six days per week, and found that women engaged in practice on four to five days per week on average (duration of practice was not stated).

## Discussion

### Summary of Key Findings

To our knowledge, this is the first published meta-analysis of the effectiveness of mindfulness-based interventions (MBIs) during the perinatal period on mindfulness and symptoms of depression, anxiety and stress. Seventeen relevant studies were identified, the great majority of which evaluated interventions carried out in the antenatal period. Study quality was limited with the majority of studies using uncontrolled designs, and with even the highest quality studies receiving a quality rating of only six out of nine. Pre-post analyses showed significant improvements following MBIs for depression, anxiety, stress and mindfulness outcomes with small to medium effect sizes (*g* = 0.36–0.51). The overall effect of the MBIs on the studies’ primary outcome or intervention target was medium in size (*g* = 0.56). In the small number of studies which included follow-up data, pre to follow-up improvements remained significant over time. Eight studies included qualitative feedback from participants and this was typically positive. Specifically, participants reported benefitting from connecting with others in a group setting, learning to stay in the present moment, learning to regulate negative responses to challenging situations, and becoming more accepting of current experiences. Completion of the mindfulness-based interventions was reasonable, with 74% of participants meeting study-defined criteria for engagement or completion where this was recorded (these criteria ranged from attending at least half of intervention sessions to attending all sessions) or a reported 20% drop-out rate. It is difficult to compare these levels of engagement with other perinatal interventions because there is considerable inconsistency in how attrition is reported, but they appear to be in line with the average drop-out rate of 23% reported for CBT for perinatal depression [17].

While these uncontrolled findings suggest that mindfulness-based interventions may show some promise in the perinatal period, pre-post analyses do not allow improvements in symptoms to be attributed with confidence to the intervention itself, since they could be due to a natural attenuation of symptoms over time or to other events in participants’ lives including their usual perinatal healthcare. Also, some studies had potentially confounding variables such as including women who were undergoing psychotherapy or taking medication at the same time as the mindfulness intervention. The eight studies that used a more robust controlled design (seven randomised-controlled designs and one non-randomised controlled trial) did not show any significant overall benefits for depression, anxiety, stress or mindfulness outcomes at post-intervention for intervention participants in comparison to controls, and effect sizes were small or negligible.

### Findings in context

#### Pre-post effects

Across all studies, this meta-analysis found moderate pre-post effects for depression (*g =* 0.47, 95% CI [0.33, 0.60]), of a magnitude comparable with those found by Hoffman et al, 2010 [26] in their meta-analysis of the effect of mindfulness on anxiety and depression in mental health and physical health populations (*g* = 0.59). For anxiety, smaller effects were identified (*g* = 0.36, 95% CI [0.17, 0.56]), and these were significantly smaller than in Hofmann et al’s [26] analyses (*g* = 0.63). In the current meta-analysis, when analysis was restricted to studies where participants were recruited on the basis of having elevated scores on measures of depression, anxiety or stress at baseline (or a history of such difficulties) pre-post effect sizes were larger (*g* = 0.61 for depression and *g* = 0.73 for anxiety) and compared favourably to Hoffman et al.’s findings. This suggests that for those who are more vulnerable to depression or anxiety, MBIs are associated with similar pre-post benefits on these outcomes during the perinatal period as those found in other clinical populations.

By contrast, when we restricted our analysis to studies with healthy or universal perinatal samples we found only small effects of MBIs on symptoms of anxiety and depression in the perinatal period (*g* = 0.35 for depression and *g* = 0.22 for anxiety). By comparison, Khoury et al [69] found moderate pre-post effects for depression (g = 0.68 (95% CI = 0.43–0.93) and anxiety (g = 0.55 (95% CI = 0.19–0.92) in a meta-analysis of MBIs for healthy, non-clinical populations. This suggests that, among healthy perinatal populations, the MBIs in the current meta-analysis may have had smaller effects than those found for MBIs in other healthy populations.

#### Between-group effects

As outlined, the current meta-analysis did not identify any significant benefits of MBIs on mindfulness or symptoms of depression, anxiety or stress in comparison to control conditions. By contrast, Hofmann et al [26] found significant between-group effect sizes for controlled studies of MBIs for depression (*g* = 0.33, p < .01) and anxiety (*g* = 0.41, p < .01) in mental health and physical health populations. Similarly, Khoury et al [69] found significant between-group effects of MBIs for depression (*g* = 0.80, p < .01), anxiety (*g* = 0.64, p < .01), and stress (*g* = 0.74, p < .01) in samples of healthy adults. This suggests that, while MBIs in the current meta-analysis did not appear to be actively unhelpful or harmful, improvements for those taking part in these interventions relative to controls were not apparent, in contrast to previous meta-analyses of MBIs in other populations.

The negligible between-group effects found in this meta-analysis appear to be less positive than findings from meta-analyses of other psychological interventions in the perinatal period. For example, in meta-analyses of the effectiveness of psychological interventions—typically Interpersonal Psychotherapy (IPT) or cognitive-behavioural therapy (CBT)—for depression during the antenatal or postnatal period, Sockol et al [17, 70] found large between-group effects of *g* = 0.96 for controlled studies of IPT and *g =* 0.40–0.65 for CBT interventions on measures of depression severity. Similarly, Cuijpers et al [18] found moderate significant between-group effects (*g =* 0.61) in a meta-analysis of the effectiveness of psychological interventions for postnatal depression. It is notable that these meta-analyses [17, 18, 70] focused on changes in symptoms of depression in participants with a diagnosis of depression. By contrast, in the current meta-analysis the majority of studies aimed to improve general wellbeing outcomes in healthy pregnant women, rather than specifically targeting those with elevated symptoms. Consequently, it may be that the effects of psychological interventions on depression, anxiety or stress in the perinatal period are only pronounced in women with significant clinical difficulties and are not noticeable in women who are already functioning well at baseline. Indeed, some authors commented that low baseline scores may have limited the scope for positive change to be seen (e.g. [34]) while, as outlined, moderation analyses of pre-post effects suggested that effect sizes were significantly larger for studies where participants were recruited on the basis of having elevated levels of anxiety, depression or stress at baseline, or of a history of mental health difficulties. However, the small number of controlled studies which did include samples of women with elevated symptoms of anxiety, stress or depression or a history of difficulties did not show significant benefits of MBIs either [35, 37, 60], suggesting factors beyond this floor effect may better explain these outcomes (although one of these studies [37] arguably focused more on preventing future depressive relapse than on reducing current symptoms).

What then might account for the negligible effects of MBIs on depression, anxiety and stress in comparison to control conditions? It is notable that the MBIs in our meta-analysis were diverse, variable with respect to their structure, aims and content and differed from the ‘gold standard’ eight session MBCT/MBSR in a number of ways. In particular, they often included fewer sessions, mindfulness practices of shorter duration and/or less frequent mindfulness practice was recommended. Moreover, the frequency and duration of mindfulness practice reported by participants was typically considerably less than recommended. Both MBCT and MBSR include a range of mindfulness practices of up to 30–40 minutes in duration. Mindfulness practices are the cornerstone of each group session and daily mindfulness practice is strongly encouraged and supported through audio recordings. Frequent mindfulness practice is purported to be fundamental to the therapeutic benefits of MBCT and MBSR [71], and it has been shown that increases in mindfulness may mediate the relationship between mindfulness practice and symptom severity and well-being outcomes [72]. It is therefore noteworthy that, although there was a small effect in favour of intervention group participants, participants in the MBI groups in the current meta-analysis did not improve significantly more than controls in their mindfulness skills. In other words, although in some cases there were significant pre-post increases in mindfulness for intervention participants but not controls, between-group differences did not reach significance (with the exception of Perez-Blasco et al’s study [34]), suggesting that the mindfulness interventions did not lead to significantly greater changes in mindfulness for intervention participants than controls. This is not consistent with findings of other meta-analyses of MBIs where between-group improvements in mindfulness are typical (e.g. [29, 73]). Therefore, a possible explanation for the lack of between-group differences on measures of depression, anxiety or stress is that there was simply insufficient time devoted to a key vehicle of therapeutic change–namely, mindfulness practice.

In addition, MBCT and MBSR include components beyond teaching mindfulness. In MBSR communication skills are taught and MBCT includes elements of cognitive therapy and behavioural activation. It may partly be for these reasons that MBSR and MBCT have larger effects than meditation interventions based purely on meditation [73]. This suggests that the full integrated intervention package, manualised in the ‘gold standard’ 8-week courses (i.e. MBCT, MBSR), may have crucial additional benefits beyond its mindfulness components. Recent research has begun to explore the ‘active ingredients’ of MBIs (e.g. [74]) and this is an important focus for future research in general as well as specifically for MBIs in the perinatal period.

In summary therefore, the apparent lack of effects on depression, anxiety and stress may be due to key differences between the MBIs included in this review and the MBCT/MBSR format rather than because MBIs are not effective in the perinatal period *per se*. Whilst additional research is needed to explore this further, it is notable that in the current meta-analysis the only study to show a significant post-intervention between-group benefit—that by Perez-Blasco et al [34]—was one of only three RCTs that included a full programme of eight 2-hour long weekly sessions based explicitly on MBSR and MBCT. In this study, although there were no significant post-intervention between-group differences in depression, the intervention group did show significantly greater improvements than controls at post intervention in stress, anxiety and mindfulness, with large effect sizes. This study included only a very small sample of breastfeeding mothers (*n* = 21), and therefore results should be treated with caution, but taken as a whole the results could suggest that researchers evaluating MBIs in the perinatal period need to give consideration to the ways in which MBIs are adapted from the standard MBCT/MBSR format to avoid potentially rendering them less effective.

Perez-Blasco et al’s [34] study was also the only study exclusively to explore the efficacy of an MBI carried out postnatally as opposed to antenatally. While it is not possible to draw conclusions based on this one study, it may be helpful to explore further whether mindfulness-based approaches could show promise for the postnatal period. Indeed, Dimidjian et al [37] also note in their study that the postnatal period appeared to present the window of greatest risk for depressive relapse, and that MBCT primarily appeared to be protective against depressive relapse postnatally. Likewise, findings of previous meta-analyses of psychological interventions designed for the perinatal period have found that interventions initiated during the postpartum period are associated with larger improvements than interventions initiated during pregnancy [17, 75]. It is possible that in some cases symptoms experienced antenatally resolve spontaneously over the course of pregnancy, whereas postnatal difficulties require greater input.

In addition to those already discussed, there are a number of important further limitations to the conclusions that can be drawn from this systematic review and meta-analysis. In particular, research exploring the effectiveness of MBIs for perinatal mental health is in its infancy, as reflected by the relatively small number of relevant studies identified. Only seventeen studies met our inclusion criteria and the overall methodological quality of these studies was poor. Only nine studies used a controlled design and the majority of these (n = 7) used a waitlist or usual care control group, which means that limited conclusions can be drawn about mechanisms of change. This is further compounded by the fact that several studies did not measure mindfulness so were unable to examine whether mindfulness mediated any positive results, and some provided little information about the training and qualifications of those who delivered the MBIs. Future research in this area should focus on improving the quality of studies to help improve the validity of their findings.

Additionally, most studies included treatment as usual comparison groups, and control group participants at times attended pregnancy groups as part of their antenatal care (such as pregnancy yoga classes), which may themselves have been beneficial, thereby reducing the potential for between-group differences to emerge. Sample sizes were also typically small, which increases the chance of pre-intervention differences existing between control and intervention group participants–although meta-analysis helps to counteract this potential problem by pooling data from multiple studies and thereby diluting the effects of pre-intervention between-group differences within individual studies across studies and correcting for biases dues to small sample sizes.

### Future research

UK clinical guidelines recommend MBCT for the prevention of depressive relapse in people with a history of depression [23], but only two studies explored this in a perinatal population [37, 56]. Both of these studies reported positive findings for MBCT for preventing depression. In particular, while Dimidjian et al’s [37] RCT data did not demonstrate a significant difference in depressive symptoms between intervention and control participants at post-intervention, there was nonetheless an estimated rate of depressive relapse of only 18.4% for intervention participants (up to six months postpartum) compared to 50.2% for control participants. Consequently, future research may benefit from further exploring the potential of MBCT for preventing perinatal depression using high quality randomised-controlled designs, exploring the role of mindfulness as a mediator of change, and preferably including an active control group. Given the small sample sizes used in most studies to date, larger more robust trials are required to help increase confidence in findings and, in particular, consideration should be given to any adaptations made from MBCT or MBSR.

As outlined, only one study to date has exclusively explored the use of mindfulness in the postnatal period. This study revealed positive findings for anxiety, stress and mindfulness, but the sample size was very small and replication with a larger sample would be beneficial. Additionally, recent UK NICE guidelines [14] emphasise the importance of examining whether interventions that focus on parent-infant interactions are beneficial for women with mental health difficulties. However, despite speculation that mindfulness-based approaches may be beneficial for early parenting [20] no studies were identified that focused on this. Consequently, this would be a valuable avenue for future research.

Although it is increasingly recognised that men are also at risk of perinatal mental health difficulties [7] only one study analysed results for expectant fathers as well as mothers. This study found borderline significant results for men compared with a control group, with medium effect sizes, but no significant effects for women. Consequently future research may benefit from further exploring mindfulness-based interventions for fathers during the perinatal period.

Finally, most studies to date have consisted of samples that are ethnically and socially homogenous, with the majority based in the US and including self-referring samples. The one study which did include harder-to-reach low-income African American women experienced high levels of attrition [36]. Future research would therefore benefit from further examining whether more diverse and representative samples benefit from mindfulness interventions and whether these populations can successfully be engaged in these interventions.

### Clinical implications

Our systematic review finds no evidence of harm on measured outcomes for mindfulness based interventions delivered in the perinatal period, and some evidence from qualitative data that they may be experienced as supportive and enjoyable. Therefore, if parents (or prospective parents) wish to access these interventions in community contexts this should not be discouraged. However, research to date does not show convincing evidence of clinical benefit from these interventions in more methodologically robust controlled studies and therefore at this stage we do not recommend offering them routinely in clinical settings, rather we recommend offering these interventions in a research context so that questions of effectiveness, moderators and mechanisms can be more fully explored. There is some evidence from our analyses of pre-post measures that people who are more vulnerable to depression or anxiety may gain more benefit from MBIs during the perinatal period than universal perinatal populations, and therefore research could focus on this at risk group for investigation in more robust studies in the first instance.

### Conclusions

This meta-analysis suggests that, whilst mindfulness-based interventions offered during the perinatal period are associated with pre- to post-intervention improvements in depression, anxiety and stress, between-group findings suggest no overall effect on these outcomes for MBI participants in comparison to control conditions. One possible reason for this could be that the MBIs in this review deviated in critical ways from full, integrated MBSR and MBCT programmes. In particular, considerably less time was spent practicing mindfulness than in MBSR or MBCT and this may have been a crucial factor contributing to limited outcomes. This suggestion is supported by the lack of between-group effects on self-reported mindfulness–that is, overall findings suggest that participants did not learn mindfulness relative to controls. Committing to regular mindfulness practice during the perinatal period may be particularly challenging given the very real demands on parents’ time and yet, findings from this review suggest that MBIs with minimal home mindfulness practice may not be of benefit to mental health in the perinatal period. This is only an emerging area however and studies to date have been diverse and heterogeneous with significant limitations, such as using small non-clinical convenience samples, and uncontrolled designs or non-active control groups, making it challenging to draw definitive conclusions. Future research should include larger more diverse and representative clinical samples, with randomised-controlled designs, and active control groups. In particular, attention should be paid to methods to enhance adherence to evidence-based MBCT and MBSR packages, intervention engagement, and to accurately record time spent practicing mindfulness.

## Supporting Information

S1 Table PRISMA Checklist. (DOC)
